# Plasma and serum volume remain unchanged following a 12-h fast from food and drink despite changes in blood and urinary hydration markers

**DOI:** 10.1038/s41430-024-01526-5

**Published:** 2024-10-17

**Authors:** Jessica E. Morgan, Olivia N. Dunning, Nicholas D. Tocci, Erica L. Mauney, Aidan S. Yazell, Matthew J. Rogatzki

**Affiliations:** Department of Public Health and Exercise Science, Appalachian State University, Boone, NC USA

**Keywords:** Biomarkers, Homeostasis, Screening

## Abstract

**Background/Objectives:**

The effect of mild dehydration on plasma and serum volume has not been well established. Furthermore, the ability of urinary and blood biomarkers to monitor small hydration changes have not been solidified. There were two objectives of this research: 1. Determine if mild dehydration affects plasma and serum volume; 2. Determine if mild dehydration can be detected better by urinary or blood biomarkers.

**Subjects/Methods:**

47 subjects were recruited; 10 subjects were removed from the study and 37 subjects (27% male) completed the study. This was a crossover study design such that each subject underwent all protocols in a counterbalanced order. Protocols consisted of 12-h dehydration, 12-h hydration, and control.

**Results:**

Neither plasma volume (*p* = 0.914), plasma volume status (*p* = 0.649), nor serum volume (*p* = 0.273) were different among protocols. Body mass (*p* < 0.001) was lower following the dehydration protocol. Urine color (*p* < 0.001), urine osmolality (*p* < 0.001), urine specific gravity (*p* < 0.001), serum osmolality (*p* < 0.001), and plasma osmolality (*p* < 0.001) were all lower following the hydration protocol. Hematocrit (*p* = 0.842) and hemoglobin concentration (*p* = 0.558) were not different among protocols.

**Conclusions:**

Dehydration did not affect plasma or serum volume. Therefore, a 12-h fast from food and water as done in this study will not likely affect laboratory test results of biomarker concentration. All 3 urinary measures were able to detect changes in hydration status, whereas only 2 blood measures were able to detect changes in hydration status. This may indicate that urinary measures are best at detecting small changes in hydration status.

## Introduction

Hydration status has been shown to affect exercise performance [1–3], cognitive performance [4, 5], and laboratory test results [6]. It is likely the effect of hydration on these factors is a result of changes in plasma volume (P_V_). The effect hydration status and exercise have on P_V_ is well understood [2, 7–10]. However, less research has investigated how mild dehydration, in the absence of exercise affects both P_V_ and serum volume (S_V_). Understanding the effects of hydration status on P_V_ and S_V_ may be important clinically, as the concentration of plasma [11–14] and serum [15–17] biomarkers are used to diagnose disease. Concentration of these biomarkers may vary based on hydration status, ultimately affecting diagnoses. For example, body mass index as an estimate of P_V_ has been shown to effect biomarkers used to assess Alzheimer’s disease [6]. Hydration status may also affect the bioavailability of water-soluble drugs [18]. Previous research has found changes in P_V_ due to hydration status [19–21] which is likely to affect S_V_, as serum is simply plasma without clotting factors [22]. Yet, we were unable to find research specifically assessing the effect of hydration status on S_V_.

While many studies use body weight as a means of assessing hydration status [23, 24], dehydration may occur with no significant changes in body weight [25]. Additionally, it may be difficult to determine changes in body weight as a result of hydration status in the clinic, where patients are wearing different clothing from previous visits or may have experienced weight losses or gains due to changes in lean and fat mass [26]. Therefore, urinary or blood measurements may be better alternatives for assessing hydration status clinically.

This study was conducted for two primary purposes: 1. To determine if dehydration causes P_V_ or S_V_ changes at rest; and 2. To determine which urinary and blood markers of hydration are able to detect dehydration in subjects adhering to protocols causing modest changes in hydration status.

## Materials/subjects and methods

### Study design

This was a crossover study design. A formal sample size calculator was used to determine that at least 30 subjects were needed to reach a power of 90% with an alpha level 0.01 [27]. Forty-seven subjects were recruited for this study and all were Caucasian. None of the subjects had comorbid heart disease, nor were any of them using diuretics. Informed consent was obtained from all subjects, and the Appalachian State University Institutional Review Board for the protection of human subjects (Boone, NC, USA) approved the study (IRB#18-0083). All subjects participated in the control, hydration, and dehydration protocols in a counterbalanced order.

**Fig. 1:**
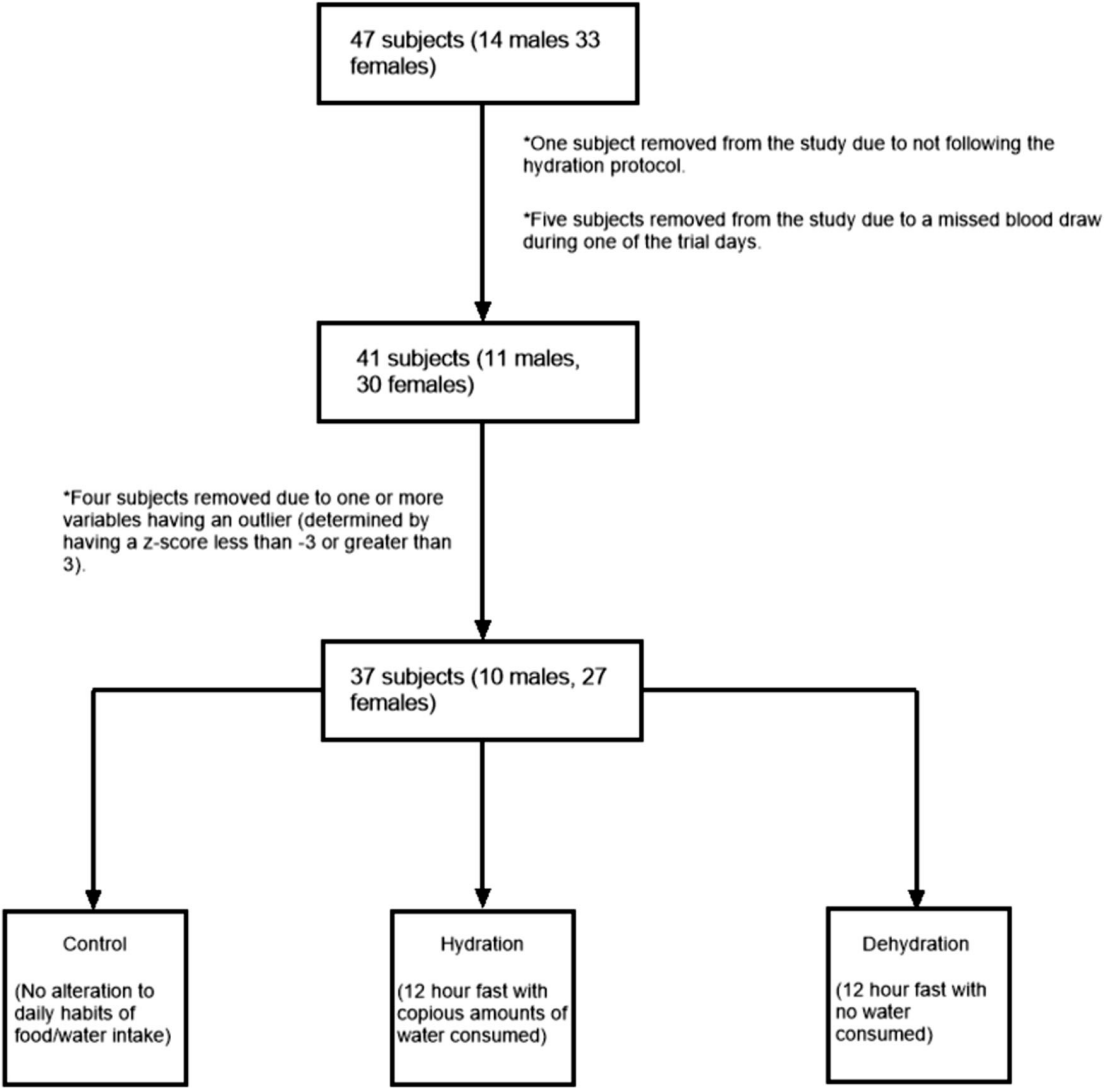
Schematic representation of data collection from initial recruitment to data used for statistical analysis.

The hydration and dehydration protocol required a 12-h fast from food. The hydration protocol required subjects to drink water during the 12-h fast (at least 6, 8-ounce (237 mL) glasses for males and at least 4, 8-ounce (237 mL) glasses for females), while the dehydration protocol required a 12-h fast from fluid ingestion. Participants were reminded via email the day before their lab visit to begin the protocol exactly 12-h before coming to lab. Twelve hours was chosen to mimic the clinical relevance of fasting 12-h prior to common blood tests. For the control protocol, subjects maintained their normal dietary routine prior to measurements. Subjects reported to lab the same time of day for all protocols to control for diurnal rhythm. Each subject was required to complete all three testing protocols within a period of 7days to minimize effects from weight changes, with a washout period of at least 24 h between protocols. Of the 47 subjects, 1 did not follow the protocol on their hydration day and 5 had issues with blood draw. Data from these 6 subjects were removed from analysis leaving a total of 41 subjects who completed the study (Fig. 1).

### Variables

To determine hydration status, the following measurements were analyzed: spot urine osmolality (U_OSM_), spot urine specific gravity (USG), spot urine color (U_C_), body mass (BM), percent total body water (%TBW), hematocrit (Hct), hemoglobin concentration ([Hb]), plasma osmolality (P_OSM_), serum osmolality (S_OSM_), plasma volume (P_V_), plasma volume status (PVS), and serum volume (S_V_). When a subject came to the laboratory, blood was drawn from a prominent vein in the antecubital space while seated [28] using a 21-gauge butterfly needle with a 7-inch luer lock extension connected to a vacutainer adapter (Becton-Dickinson, Franklin Lakes, NJ USA). Blood was collected into a 4 ml heparin and a 4 ml serum separation vacutainer (Becton-Dickinson, Franklin Lakes, NJ USA).

After blood collection, the subject entered a private restroom where he/she provided a urine sample, emptied his/her bladder completely, and then performed a naked body weight and %TBW measurement using the Tanita BC-533 bioelectrical impedance (BIA) scale (Tanita Corporation of America, Inc., Arlington Heights, IL USA). This scale was used due to its ability to give repeatable weight and %TBW measurements, as tested in-house prior to commencement of the study. This type of scale does not distinguish between intracellular water and extracellular water. The urine sample was not a first morning sample as first morning urine may not strongly correlate with fluid intake [29].

The urine sample was visually analyzed for color using the sample-over-chart method [30] in ambient fluorescent laboratory lighting by the same non-blinded investigator [31]. Urine Osmolality was measured using a single sample osmometer (Advanced Instruments Model 3250, Norwood, MA USA). Specific gravity was measured using an analog handheld refractometer (ATAGO U.S.A., Inc., Bellevue, WA USA).

Blood from the heparin vacutainer was used to determine Hct in duplicate (1.32% intra-assay coefficient of variation) by filling two microhematocrit tubes with blood from the heparinized vacutainer, which were then centrifuged at 13,700 × *g* for two minutes using a microhematocrit centrifuge (StatSpin CritSpin, HemoCue America, Brea, CA USA). Hematocrit was determined by measuring the height of blood and plasma divided by the height of red blood cells (RBCs) and multiplied by 100. A 10-fold dilution was performed by placing 1 ml of blood from the heparinized vacutainer into 9 ml of deionized water (dH_2_O) for 30 min to allow lysing of RBCs. Following this incubation period a 10-fold series dilution was performed two more times using dH_2_O to obtain a final 1000-fold dilution. This 1000-fold dilution was used to determine [Hb] via the Harboe method with an Allen correction factor [32]. Briefly, the 1000-fold diluted sample was poured into a cuvette and underwent spectrophotometry at the wavelengths of 380, 415, and 450 nanometers (nm) using an Eon spectrophotometer (BioTek Instruments, Inc., Winooski, VT USA). The absorbance at each wavelength, after subtraction from a dH_2_O blank, were used to determine [Hb] with a modified version of the Harboe equation to give hemoglobin results in g/dl instead of mg/dl. Thus, the following equation was used: Hb (g/dl) = ((0.01672 x A_415_) – (0.00836 x A_380_) – (0.00836 x A_450_)) * 1,000; where 0.01672 and 0.00836 are constants, A_415_ represents absorbance at 415 nm, A_380_ represents absorbance at 380 nm, A_450_ represents absorbance at 450 nm, and multiplying by 1,000 accounts for the 1,000-fold dilution.

Hematocrit and [Hb] were then used to determine plasma volume using the following equations developed by Dill and Costill, 1974 [7]:

$${{\rm{BV}}}_{{\rm{A}}}={{\rm{BV}}}_{{\rm{B}}}({{\rm{Hb}}}_{{\rm{B}}}/{{\rm{Hb}}}_{{\rm{A}}})$$

$${{\rm{CV}}}_{{\rm{A}}}={{\rm{BV}}}_{{\rm{A}}}({{\rm{Hct}}}_{{\rm{A}}})$$

$${{\rm{PV}}}_{{\rm{A}}}={{\rm{BV}}}_{{\rm{A}}}-{{\rm{CV}}}_{{\rm{A}}}$$

$${{\rm{CV}}}_{{\rm{B}}}={{\rm{BV}}}_{{\rm{B}}}({{\rm{Hct}}}_{{\rm{B}}})$$

$${{\rm{PV}}}_{{\rm{B}}}={{\rm{BV}}}_{{\rm{B}}}-{{\rm{CV}}}_{{\rm{B}}}{\rm{;}}$$

where BV = blood volume, Hb = hemoglobin, CV = red blood cell volume, Hct = hematocrit, PV = Plasma Volume, _A_ = after dehydration, _B_ = before dehydration, BV_B_ was considered to be 100.

Plasma volume was calculated with control values for [Hb] and Hct entered in the “before” part of the formulas and [Hb] and Hct values for dehydration or hydration put in the “after” part of the formulas to provide consistency of the P_V_ calculations.

Plasma volume status has also been shown to be an accurate measure to assess plasma volume [33, 34]. Therefore, PVS was calculated according to Hoshika et al. [33]:

$${\rm{Actual}}\; {\rm{PV}}=(1-{\rm{hematocrit}})* \left[{\rm{a}}+({{\rm{b}}}* {\rm{body}}\; {\rm{weight}}\; {\rm{in}}\; {\rm{kg}})\right.$$

$${\rm{Ideal}}\; {\rm{PV}}={{\rm{c}}}*{\rm{body}}\; {\rm{weight}}\; {\rm{in}}\; {\rm{kg}}$$

$${\rm{PVS}}( \% )={[({\rm{actual}}\; {\rm{PV}}-{\rm{ideal}}\; {\rm{PV}})/{\rm{ideal}}\; {\rm{PV}}]}*100;$$

where PV = plasma volume, a = 1530 in males and 864 in females, b = 41 in males and 47.9 in females, kg = kilograms, c = 39 in males and 40 in females, PVS = plasma volume status.

The remaining 3 mL of blood in the heparin vacutainer underwent centrifugation at 2630 × *g* for 10 min at 4 °C (ThermoScientific Sorvall Legend RT+ refrigerated centrifuge, Thermo Fisher Scientific, Inc., Walthum, MA USA) to separate the plasma. Following centrifugation, P_OSM_ was determined via the single-sample osmometer.

3 mL of blood were removed from the serum separation vacutainer and placed into a serum transfer tube. This was done to ensure the same amount of blood was used to determine S_V_ for each sample, as no formula exists for calculating S_V_. The 3 mL of blood was then allowed to coagulate at room temperature for greater than 30 min, but no longer than 60 min. Following coagulation, the blood underwent centrifugation at 2630 × *g* for 10 min at 4 °C. The separated serum was then poured into a graduated cylinder standing on a chemical scale (Mettler Toledo XS104, Mettler-Toledo, LLC, Columbus, OH USA) to determine S_V_ produced per 3 mL of blood both by visual measurement using the graduated cylinder and by weighing the serum sample with the assumption that 1 µl of serum has a mass of 1 mg. Once the measurement of S_V_ was complete, S_OSM_ was measured via freezing point depression using the single sample osmometer. All measurements were performed immediately following blood and urine collection to avoid any fluid changes due to storage [35].

### Statistical analysis

One subject forgot to measure %TBW on their control day creating one missing data point. To account for this, a multiple imputation approach using 5 imputations and taking the mean was used. The original data set of %TBW was then compared to the %TBW data following multiple imputation using a two-tailed paired *T*-test. Outliers for all variables were identified using the Z score method with cutoff points of 3.0 and −3.0. This decision was made a priori in order to remove subjects who likely did not comply with one of the protocols.

A repeated measures analysis of variance (RMANOVA) was used to compare all variables when multivariate normality was met. When Mauchley’s test of sphericity was not met, a Greenhouse–Geisser Correction factor was used. For variables in which multivariate normality was not met, a two-way Friedman’s non-parametric test was used. Bonferroni post-hoc analyses were used when significance was found to determine differences among variables. Partial eta squared (η^2^) was used to calculate effect size when RMANOVA was used, and Kendall’s W was used for a Friedman test.

To determine sex differences between age, a two-tailed independent samples *T*-test was conducted when the assumption of normality and homogeneity of variance for mean was met. A two-tailed Mann–Whitney *U* test was conducted when normality or homogeneity of variance for mean was not met. Cohen’s D (d) was used to calculate effect size when a two-tailed independent samples *T*-test was conducted and η^2^ was used to calculate effect size when a Mann–Whitney *U* test was conducted.

Sex differences for all other variables were compared using a multivariate ANOVA (MANOVA) with Pillai’s trace even if normality was violated, as long as homogeneity of variance was met, as the MANOVA has been found to outperform the nonparametric test when only the assumption of normal distribution is violated [36]. If the assumption of homogeneity of variance was violated, a nonparametric multivariate Kruskal–Wallis test was used.

Normality was tested using a Shapiro–Wilk Normality test. Homogeneity of variance was tested using a Levene statistic. Alpha was set at 0.05 to determine statistical significance. All statistical analyses were generated using the Statistical Package for the Social Sciences (SPSS) version 28 (SPSS Inc., Chicago, IL USA).

## Results

The paired *T*-test between the original %TBW data set and the %TBW data set following multiple imputation indicated a standard deviation of zero. Therefore, we used the data set derived from multiple imputation for all statistical analyses. The Z score method showed 4 subjects with outliers. These 4 subjects were removed giving a total of 37 subjects (10 males and 27 females) for statistical analysis (Fig. 1).

Normality was met in all protocols for %TBW and S_OSM_ (*p* > 0.05) but not for BM, age, %TBW, U_C_, USG, U_OSM_, Hct, [Hb], P_V_, PVS, P_OSM_, S_V_ in grams, or S_V_ in milliliters (*p* < 0.05). Homogeneity of variance based on mean for all protocols was met for each variable (*p* > 0.05) except USG and U_OSM_ (*p* < 0.05). Normality based on sex was met for P_V_, PVS, and S_OSM_ (*p* > 0.05) but not for any other variable (*p* < 0.05). Homogeneity of variance based on mean for sex was met in all variables (*p* > 0.05).

There were no differences among protocols for %TBW or age (*p* = 1.000). Serum osmolality was lower in the hydration group compared to the dehydration (*p* < 0.001) and control groups (*p* < 0.001). There were no significant differences among protocols for Hct (*p* = 0.842), [Hb] (*p* = 0.558), P_V_ (*p* = 0.914), PVS (*p* = 0.649), S_V_ in grams (*p* = 0.273), or S_V_ in milliliters (*p* = 0.334).

**Fig. 2:**
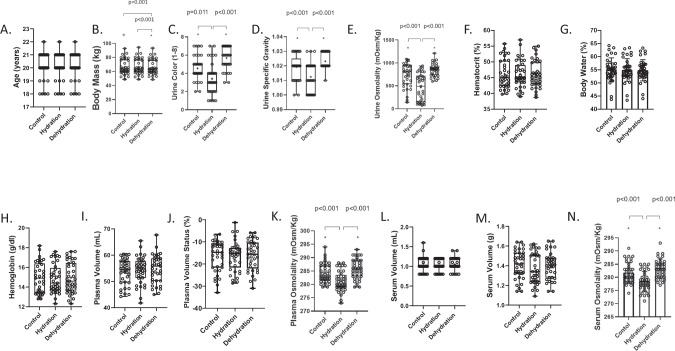
Graphs showing differences in the variables of age. **A** Body mass (**B**), urine color (**C**), urine specific gravity (**D**), urine osmolality (**E**), hematocrit (**F**), percent body water (**G**), hemoglobin concentration (**H**), plasma volume (**I**), plasma volume status (**J**), plasma osmolality (**K**), serum volume in milliliters (**L**), serum volume in grams (**M**), and serum osmolality (**N**) among the control, hydration, and dehydration protocols. Box and whisker plots are used to represent nonparametric data where the ‘+’ represents the mean value and the line through the box represents the median value. Interquartile ranges were calculated using inclusive median. Bar graphs are used to represent parametric data with standard deviation. * = significantly different compared to the hydration protocol (*p* < 0.05). + = significantly different compared to the dehydration protocol (*p* < 0.05).

Body mass was 0.14% lower following dehydration compared to control (*p* = 0.001) and 0.86% lower than the hydration protocol (*p* < 0.001). Urine color was lower following the hydration protocol compared to both the dehydration (*p* < 0.001) and control (*p* = 0.011) protocols. Urine osmolality, USG, and P_OSM_, were all lower following the hydration protocol compared to the dehydration (*p* < 0.001) and control (*p* < 0.001) protocols. See Fig. 2 and Supplementary Data 1 for comparisons among protocols.

### Sex differences

**Fig. 3:**
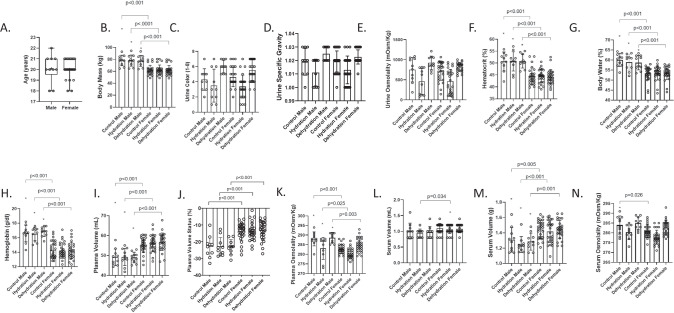
Graphs showing the differences between males and females for the variables of age. **A**, body mass (**B**), urine color (**C**), urine specific gravity (**D**), urine osmolality (**E**), hematocrit (**F**), percent body water (**G**), hemoglobin concentration (**H**), plasma volume (**I**), plasma volume status (**J**), plasma osmolality (**K**), serum volume in milliliters (**L**), serum volume in grams (**M**), and serum osmolality (**N**) among the control, hydration, and dehydration protocols. Box and whisker plots are used to represent nonparametric data where the ‘+’ represents the mean value and the line through the box represents the median value. Interquartile ranges were calculated using inclusive median. Bar graphs are used to represent parametric data with standard deviation. * = significantly different compared to males (*p* < 0.05).

Body mass, %TBW, Hct, and [Hb], were greater in males compared to females for all protocols (*p* < 0.001). Plasma osmolality was also greater in males for the control (*p* < 0.001), hydration (*p* = 0.025), and dehydration (*p* = 0.003) protocols. Males had higher S_OSM_ but only at the control protocol (*p* = 0.026). On the other hand, females had a greater P_V_ and PVS in all protocols (*p* < 0.001). Serum volume in grams was greater in females for the control (*p* = 0.005), hydration (*p* < 0.001), and dehydration (*p* = 0.001) protocols. Females also had a higher S_V_ in milliliters but only at the hydration protocol (*p* = 0.034). There were no differences between sexes for age, U_C_, USG, or U_OSM_ (*p* > 0.05). See Fig. 3 and supplementary Data 2 for comparison between sexes.

## Discussion

There were 2 purposes of this study: 1. To determine if a 12-h fast affects P_V_ or S_V_; 2. To analyze the performance of blood and urinary markers in detecting hydration changes. In regard to the first purpose, we found that neither P_V_ nor S_V_ were affected by the dehydration and hydration protocols. For the second purpose, we found that BM, S_OSM_, P_OSM_, U_C_, U_OSM_, and USG did show differences among protocols.

The finding that P_V_ and S_V_ did not change supports the practice of not considering hydration status when analyzing clinical blood tests. Hematocrit and [Hb] also did not change, which is expected given the calculation used to determine P_V_ is dependent upon Hct and [Hb] [7]. A lack of change in Hct, [Hb], and P_V_ among our protocols is in agreement with other studies [37–41] and supports the hypothesis that P_V_ and S_V_ are maintained despite small changes in hydration status [39].

Plasma osmolality and S_OSM_ were all lower after the hydration protocol compared to the dehydration and control protocols. Our results agree with those of others who have found changes in P_OSM_ and S_OSM_ with changes in hydration status [20, 37, 40, 42–44]. However, none of the protocols in our study caused P_OSM_ or S_OSM_ to go outside of the normal range (≥290 mOsm) [1] for a hydrated individual. This is to be expected with our modest protocol, as P_OSM_ and S_OSM_ have been reported to be well controlled in light of hydration status [35, 42].

Urine osmolality, USG, and U_C_ were all lower after the hydration protocol compared to the dehydration and control protocols. All 3 urinary measures were in the dehydration category of U_OSM_≥700 mOsmol, USG≥1.020, and U_C_≥4 [4, 31, 45] following both the control and dehydration protocols. This shows that solute concentration in urine is sensitive to modest changes in fluid intake. Our finding of changes in U_OSM_ and USG with only a 0.86% reduction in BM disagree with those of Popowski et al. who found that U_OSM_ was not changed significantly until a 5% decrease in BM; and that USG was not changed significantly until a 3% decrease in BM [43]. This distinction is likely due to differences in protocols, as Popowski et al. was investigating acute dehydration caused by exercise, whereas our study employed a 12-h period of dehydration. Therefore, it is possible the urinary values did not have time to change in the Popowski study. Other research agrees with our study, supporting the use of urine measurements as an indicator of hydration status even in cases of mild hypohydration [38, 39]. Our findings of urinary measures changing with hydration status despite no changes in S_V_ or P_V_ agree with the findings of Tucker et al. who found that neither U_OSM_, USG, nor U_C_ is predictive of plasma volume [46].

Although not the primary purpose of this study, we compared all data between sexes. BM, %TBW, Hct, and [Hb] were all significantly higher in males, which has been shown previously [38, 47–49]. A unique result was that P_V_ and S_V_ was greater in females for all protocols. This finding was unexpected since males tend to have greater blood volume than females [50], and would therefore be expected to have higher P_V_ and S_V_. However, it is possible the menstrual cycle may have caused this difference, as women have reported retaining water at different times throughout the menstrual cycle [51]. In expectation from our findings on P_V_ and S_V_, P_OSM_ and S_OSM_ was found to be greater in males for all protocols. Urinary markers were not different between sexes.

There are limitations to be considered when interpreting data from this study. One is that water ingestion was not normalized by BM in the hydration protocol. This could cause less hydration in larger individuals and greater hydration in smaller individuals. Another limitation is that we did not require subjects to report the amount of fluid they ingested during the hydration protocol which would have allowed us to normalize data based on the amount of water each subject ingested. Although our data was within the normal ranges expected, we used single measures for U_OSM_, S_OSM_, P_OSM_, USG, S_V_, P_V_, and [Hb]. While we did use duplicate measures for Hct, we should have used duplicate measures for all variables tested. A final limitation to consider is that all subjects in this study were young (18–22 yrs.) and apparently healthy. Since hydration status may be affected by age and disease, the results of this study should not be extrapolated to older or diseased populations.

In conclusion, no differences were found in P_V_ or S_V_. Since P_V_ and S_V_ were not affected by these protocols, it stands to reason the concentration of clinical biomarkers would also not be affected by a 12-h fasting protocol as typically prescribed prior to common blood tests. Of the variables used to assess hydration status, the urinary variables appeared to be better at detecting small changes in hydration status compared to blood variables. Sex differences existed between BM, Hct, and [Hb] as expected. Unexpectedly, females had higher P_V_ and S_V_ than males. Future research may consider investigating an older population who more commonly undergo a 12-h fast prior to blood tests, normalize fluid based on BM, take multiple blood and urinary measures during the 12-hour period, and control for the menstrual cycle in women.

## Supplementary information

Supplementary Tables 1 & 2

Supplementary Table 3

## Data Availability

Data analyzed during this study can be found in the Supplementary Data 3 excel spreadsheet.

## References

[1] Sawka MN, Burke LM, Eichner R, Maughan RJ, Montain SJ, Stachenfeld NS. Exercise and fluid replacement. Med Sci Sports Exerc. 2007;39:377–90. 17277604 10.1249/mss.0b013e31802ca597

[2] James LJ, Moss J, Henry J, Papadopoulou C, Mears SA. Hypohydration impairs endurance performance: a blinded study. Physiol Rep. 2017;5:1–10. 10.14814/phy2.13315 PMC5492205 28637708

[3] Barley OR, Iredale F, Chapman DW, Hopper A, Abbiss CR. Repeat effort performance is reduced 24 h after acute dehydration in mixed martial arts athletes. J Strength Cond Res. 2018;32:2555–61. 28930879 10.1519/JSC.0000000000002249

[4] Thomas DT, Erdman KA, Buke LM. Nutrition and Athletic Performance. Med Sci Sports Exerc. 2016;48:543–68. 10.1249/MSS.0000000000000852 26891166

[5] Cian C, Koulmann N, Barraud PA, Raphel C, Jimenez C, Melin B. Influence of variations in body hydration on cognitive function: effect of hyperhydration, heat stress, and exercise-induced dehydration. 2000;4:29–36.

[6] Tristão‐Pereira C, Fuster V, Sanchez‐Gonzalez J, Ibañez B, Zetterberg H, Blennow K, et al. Physiological confounders of blood‐based Alzheimer’s disease biomarkers in middle‐aged asymptomatic individuals. Alzheimers Dement. 2023;19:e078219.

[7] Dill DB, Costill DL. Calculation of percentage changes in volumes of blood, plasma, and red cells in dehydration. J Appl Physiol. 1974;37:247–8. 4850854 10.1152/jappl.1974.37.2.247

[8] Jimenez C, Melin B, Koulmann N, Allevard AM, Launay JC, Savourey G. Plasma volume changes during and after acute variations of body hydration level in humans. Eur J Appl Physiol. 1999;80:1–8. 10.1007/s004210050550 10367716

[9] Graham MR, Pates J, Davies B, Cooper SM, Bhattacharya K, Evans PJ, et al. Should an increase in cerebral neurochemicals following head kicks in full contact karate influence return to play? Int J Immunopathol Pharm. 2015;28:539–46. 10.1177/0394632015577045 25816397

[10] Graham MR, Myers T, Evans P, Davies B, Cooper SM, Bhattacharya K, et al. Direct hits to the head during amateur boxing is associated with a rise in serum biomarkers for brain injury. Int J Immunopathol Pharm. 2011;24:119–25. 10.1177/039463201102400114 21496394

[11] Aaron RE, Tian T, Fleming GA, Sacks DB, Januzzi JL. Emerging biomarkers in the laboratory and in practice: a novel approach to diagnosing heart failure in diabetes. J Diabetes Sci Technol. 2024;18:733–40. 10.1177/19322968241227898 PMC11089856 38292004

[12] Yu X, Sun Y, Zhao Y, Zhang W, Yang Z, Gao Y, et al. Prognostic value of plasma galectin-3 levels in patients with coronary heart disease and chronic heart failure. Int Heart J. 2015;56:314–8. 25902879 10.1536/ihj.14-304

[13] Latini R, Masson S, Anand IS, Missov E, Carlson M, Vago T, et al. Prognostic value of very low plasma concentrations of troponin T in patients with stable chronic heart failure. Circulation. 2007;116:1242–9. 17698733 10.1161/CIRCULATIONAHA.106.655076

[14] Feola M, Testa M, Leto L, Cardone M, Sola M, Rosso GL. Role of galectin-3 and plasma B type-natriuretic peptide in predicting prognosis in discharged chronic heart failure patients. Medicine (Baltimore). 2016;95:e4014. 27368017 10.1097/MD.0000000000004014 PMC4937931

[15] Williams‐Gray CH, Wijeyekoon R, Yarnall AJ, Lawson RA, Breen DP, Evans JR, et al. Serum immune markers and disease progression in an incident P arkinson’s disease cohort (ICICLE‐PD). Mov Disord. 2016;31:995–1003. 26999434 10.1002/mds.26563 PMC4957620

[16] Sahab ZJ, Semaan SM, Sang Q-XA. Methodology and applications of disease biomarker identification in human serum. Biomark Insights. 2007;2:117727190700200. PMC2717826 19662190

[17] Ayrignac X, Le Bars E, Duflos C, Hirtz C, Maleska Maceski A, Carra-Dallière C, et al. Serum GFAP in multiple sclerosis: correlation with disease type and MRI markers of disease severity. Sci Rep. 2020;10:10923. 32616916 10.1038/s41598-020-67934-2 PMC7331703

[18] Rastmanesh R. Inadequate hydration status of test subjects can affect bioavailability studies. ACS Pharm Transl Sci. 2023;6:1104–6. 10.1021/acsptsci.3c00089 PMC10353055 37470021

[19] Patterson SM, VanderKaay MM, Shanholtzer BA, Patterson CA. Influence of acute fluid loading on stress-induced hemoconcentration and cardiovascular reactivity. J Behav Med. 2008;31:319–30. 18607711 10.1007/s10865-008-9162-7

[20] Endo Y, Torii R, Yamazaki F, Sagawa S, Yamauchi K, Tsutsui Y, et al. Water drinking causes a biphasic change in blood composition in humans. Pflüg Arch. 2001;442:362–8. 10.1007/s004240100555 11484766

[21] Nose H, Mack GW, Shi XR, Nadel ER. Role of osmolality and plasma volume during rehydration in humans. J Appl Physiol. 1988;65:325–31. 3403476 10.1152/jappl.1988.65.1.325

[22] Hrubec TC, Whichard JM, Larsen CT, Pierson FW. Plasma versus serum: specific differences in biochemical analyte values. J Avian Med Surg. 2002;16:101–5.

[23] Armstrong LE. Assessing hydration status: the elusive gold standard. J Am Coll Nutr. 2007;26:575S–84S. 17921468 10.1080/07315724.2007.10719661

[24] Kavouras SA. Assessing hydration status. Curr Opin Clin Nutr Metab Care. 2002;5:519–24. 12172475 10.1097/00075197-200209000-00010

[25] Hahn RG. Effects of diet, habitual water intake and increased hydration on body fluid volumes and urinary analysis of renal fluid retention in healthy volunteers. Eur J Nutr. 2021;60:691–702. 32430554 10.1007/s00394-020-02275-4 PMC7900032

[26] Kavouras SA. Hydration, dehydration, underhydration, optimal hydration: are we barking up the wrong tree? Eur J Nutr. 2019;58:471–3. 30607564 10.1007/s00394-018-01889-z

[27] Faul F, Erdfelder E, Lang A-G, Buchner AG. Power 3: a flexible statistical power analysis program for the social, behavioral, and biomedical sciences. Behav Res Methods. 2007;39:175–91. 17695343 10.3758/bf03193146

[28] Harrison MH. Effects on thermal stress and exercise on blood volume in humans. Physiol Rev. 1985;65:149–209. 3880897 10.1152/physrev.1985.65.1.149

[29] Perrier, Rondeau E, Poupin P, Le Bellego M, Armstrong LE L, Lang F, et al. Relation between urinary hydration biomarkers and total fluid intake in healthy adults. Eur J Clin Nutr. 2013;67:939–43. 23695204 10.1038/ejcn.2013.93 PMC3778844

[30] Wardenaar F, Armistead S, Boeckman K, Butterick B, Youssefi D, Thompsett D, et al. Validity of urine color scoring using different light conditions and scoring techniques to assess urine concentration. J Athl Train. 2022;57:191–8. 35201303 10.4085/1062-6050-0389.21 PMC8876881

[31] Fink HH, Mikesky AE. Practical applications in sports nutrition. 5th ed. Jones & Bartlett Learning; 2018.

[32] Han V, Serrano K, Devine DV. A comparative study of common techniques used to measure haemolysis in stored red cell concentrates. Vox Sang. 2010;98:116–23. 19719459 10.1111/j.1423-0410.2009.01249.x

[33] Hoshika Y, Kubota Y, Mozawa K, Tara S, Tokita Y, Yodogawa K, et al. Effect of empagliflozin versus placebo on plasma volume status in patients with acute myocardial infarction and type 2 diabetes mellitus. Diabetes Ther. 2021;12:2241–8. 34236577 10.1007/s13300-021-01103-0 PMC8342682

[34] Ling HZ, Flint J, Damgaard M, Bonfils PK, Cheng AS, Aggarwal S, et al. Calculated plasma volume status and prognosis in chronic heart failure. Eur J Heart Fail. 2015;17:35–43. 25469484 10.1002/ejhf.193

[35] Bohnen N, Terwel D, Markerink M, Ten Haaf JA, Jolles J. Pitfalls in the measurement of plasma osmolality pertinent to research in vasopressin and water metabolism. Clin Chem. 1992;38:2278–80. 1424124

[36] Finch H. Comparison of the performance of nonparametric and parametric MANOVA test statistics when assumptions are violated. Methodology. 2005;1:27–38.

[37] Stookey JD, Hamer J, Killilea DW. Change in hydration indices associated with an increase in total water intake of more than 0.5 L/day, sustained over 4 weeks, in healthy young men with initial total water intake below 2 L/day. Physiol Rep. 2017;5:e13356. 29150589 10.14814/phy2.13356 PMC5704074

[38] Armstrong LE, Maresh CM, Castellani JW, Bergeron MF, Kenefick RW, LaGasse KE, et al. Urinary indices of hydration status. Int J Sport Nutr. 1994;4:265–79. 7987361 10.1123/ijsn.4.3.265

[39] Francesconi RP, Hubbard RW, Szlyk PC, Schnakenberg D, Carlson D, Leva N, et al. Urinary and hematologic indexes of hypohydration. J Appl Physiol. 1987;62:1271–6. 3571082 10.1152/jappl.1987.62.3.1271

[40] Williams TD, Seckl JR, Lightman SL. Dependent effect of drinking volume on vasopressin but not atrial peptide in humans. Am J Physiol-Regul Integr Comp Physiol. 1989;257:R762–4. 10.1152/ajpregu.1989.257.4.R762 2529782

[41] Geelen G, Keil LC, Kravik SE, Wade CE, Thrasher TN, Barnes PR, et al. Inhibition of plasma vasopressin after drinking in dehydrated humans. Am J Physiol-Regul Integr Comp Physiol. 1984;247:R968–71. 10.1152/ajpregu.1984.247.6.R968 6507654

[42] Armstrong LE. Hydration assessment techniques. Nutr Rev. 2005;63:S40–54. 16028571 10.1111/j.1753-4887.2005.tb00153.x

[43] Popowski LA, Oppliger RA, Patrick Lambert G, Johnson RF, Kim Johnson A, Gisolfi CV. Blood and urinary measures of hydration status during progressive acute dehydration. Med Sci Sports Exerc. 2001;33:747–53. 11323543 10.1097/00005768-200105000-00011

[44] Moran DS, Heled Y, Margaliot M, Shani Y, Laor A, Margaliot S, et al. Hydration status measurement by radio frequency absorptiometry in young athletes—a new method and preliminary results. Physiol Meas. 2004;25:51–9. 15005304 10.1088/0967-3334/25/1/005

[45] Kavouras SA, Johnson EC, Bougatsas D, Arnaoutis G, Panagiotakos DB, Perrier E, et al. Validation of a urine color scale for assessment of urine osmolality in healthy children. Eur J Nutr. 2016;55:907–15. 25905541 10.1007/s00394-015-0905-2 PMC4819932

[46] Tucker MA, Butts CL, Satterfield AZ, Six A, Johnson EC, Ganio MS. Spot sample urine specific gravity does not accurately represent small decreases in plasma volume in resting healthy males. J Am Coll Nutr. 2018;37:17–23. 28985131 10.1080/07315724.2017.1323692

[47] Malisova O, Athanasatou A, Pepa A, Husemann M, Domnik K, Braun H, et al. Water intake and hydration indices in healthy European adults: the European hydration research study (EHRS). Nutrients. 2016;8:1–12. 10.3390/nu8040204 PMC4848673 27058557

[48] Weitkunat T, Knechtle B, Knechtle P, Rüst CA, Rosemann T. Body composition and hydration status changes in male and female open-water swimmers during an ultra-endurance event. J Sports Sci. 2012;30:1003–13. 22554315 10.1080/02640414.2012.682083

[49] Ritz P, Vol S, Berrut G, Tack I, Arnaud MJ, Tichet J. Influence of gender and body composition on hydration and body water spaces. Clin Nutr. 2008;27:740–6. 18774628 10.1016/j.clnu.2008.07.010

[50] Sharma R, Sharma S. Physiology, Blood Volume. Treasure Island, FL: StatPearls Publishing; 2023. 30252333

[51] White CP, Hitchcock CL, Vigna YM, Prior JC. Fluid retention over the menstrual cycle: 1-year data from the prospective ovulation cohort. Obstet Gynecol Int. 2011;2011:1–7. 10.1155/2011/138451 PMC3154522 21845193

