# Supplementary material for: Plasma and serum volume remain unchanged following a 12-h fast from food and drink despite changes in blood and urinary hydration markers
Source: Eur J Clin Nutr. 2024 Oct 17;79(2):161–7. doi: 10.1038/s41430-024-01526-5 (PMC11810769; doi:10.1038/s41430-024-01526-5)
Supplement: Supplementary file 1 — Supplementary Tables 1 & 2 [file 41430_2024_1526_MOESM1_ESM.docx]

| **RMANOVA** | **F** | **η^2^** | **Control** | **Hydration** | **Dehydration** |
| --- | --- | --- | --- | --- | --- |
| **Body Water (%)** | 10.162 | 0.155 | 55.15(4.42) | 54.93 (4.31) | 54.82 (4.22) |
| **Serum Osmolality (mOsmoles/Kg)** | 10.162 | 0.158 | 281.80 (3.86)**^*^** | 278.51 (3.75)**^^#^** | 283.34 (3.60)**^*^** |
| **Friedman** | **Test Statistic** | **W** | **Control** | **Hydration** | **Dehydration** |
| **Body Mass (kg)** | 20.540 | 0.278 | 63.45(59.59, 77.00)**^^^** | 63.91 (59.73, 76.91)**^^^** | 63.36 (58.82, 76.37)**^#*^** |
| **Age (years)** | 0.000 | 0.000 | 20.00(20.00, 21.00) | 20.00(20.00, 21.00) | 20.00(20.00, 21.00) |
| **Urine Color (1-8)** | 32.968 | 0.446 | 4.00 (4.00, 5.00)**^*^** | 3.00 (2.00, 4.00)**^^#^** | 6.00 (5.00, 7.00)**^*^** |
| **Urine Osmolality (mOsm/Kg)** | 36.162 | 0.489 | 807.00 (539.50, 942.00)**^*^** | 492.00 (143.00, 713.00)**^^#^** | 860.00 (738.00, 906.50)**^*^** |
| **Urine Specific Gravity** | 26.196 | 0.354 | 1.02 (1.02, 1.02)**^*^** | 1.01 (1.00, 1.20)**^^#^** | 1.02 (1.02, 1.03)**^*^** |
| **Hematocrit (%)** | 0.345 | 0.005 | 45.00 (42.38, 50.38) | 45.00 (43.50, 49.00) | 45.25 (43.00, 49.88) |
| **Hemoglobin (g/dl)** | 1.167 | 0.016 | 14.90 (13.40, 16.40) | 14.40 (13.70, 15.94) | 14.6 (13.65, 16.45) |
| **Plasma Volume (mL)** | 0.181 | 0.002 | 54.50 (49.63, 57.75) | 55.00 (51.33, 57.79) | 55.81 (50.23, 58.76) |
| **Plasma Volume Status** | 0.865 | 0.012 | -14.74 (-21.63, -11.13) | -15.32 (-20.73, -12.87) | -15.60 (-21.32, -10.55) |
| **Plasma Osmolality (mOsm/Kg)** | 33.825 | 0.457 | 283.00 (282.00, 287.00)**^*^** | 280.00 (279.00, 283.00)**^^#^** | 286.00 (283.00, 289.00)**^*^** |
| **Serum Volume (mL)** | 2.595 | 0.035 | 1.42 (1.32, 1.53) | 1.34 (1.24, 1.51) | 1.41 (1.28, 1.48) |
| **Serum Volume (g)** | 2.194 | 0.030 | 1.00 (1.00, 1.20) | 1.00 (1.00, 1.20) | 1.00 (1.00, 1.20) |

**Supplementary Table 1:** Descriptive statistics for percent body water, serum osmolality, body mass, age, urine color, urine osmolality, urine specific gravity, hematocrit, hemoglobin, plasma volume, plasma volume status, plasma osmolality, serum volume in milliliters, and serum volume in grams among the control, hydration, and dehydration protocols. Data presented as mean (SD) for parametric data and median (Q1, Q3) for nonparametric data. * = significantly different compared to hydration (p<0.05), # = significantly different compared to control (p<0.05), ^ = significantly different compared to dehydration (p<0.05)

| **Variable** | **Protocol** | **F** | **η^2^** | **Male** | **Female** | **p-value** |
| --- | --- | --- | --- | --- | --- | --- |
| **Body Water (%)** | Control | 28.428 | 0.213 | 60.04 (3.27) | 53.34 (3.27) | <0.001***** |
|  | Hydration | 18.906 | 0.153 | 58.92 (3.98) | 53.46 (3.45) | <0.001***** |
|  | Dehydration | 17.560 | 0.143 | 58.66 (3.60) | 53.39 (3.21) | <0.001***** |
| **Body Mass (kg)** | Control | 27.818 | 0.209 | 78.14 (8.69) | 62.21 (7.76) | <0.001***** |
|  | Hydration | 26.205 | 0.200 | 77.85 (9.20) | 62.40 (8.00) | <0.001***** |
|  | Dehydration | 26.314 | 0.200 | 77.38 (9.06) | 61.89 (7.82) | <0.001***** |
| **Age (years)** | Control | 124**^#^** | 0.005 | 20.00(19.50, 21.00) | 20.00 (20.00, 21.00) | 0.658 |
|  | Hydration | 124**^#^** | 0.005 | 20.00(19.50, 21.00) | 20.00 (20.00, 21.00) | 0.658 |
|  | Dehydration | 124**^#^** | 0.005 | 20.00(19.50, 21.00) | 20.00 (20.00, 21.00) | 0.658 |
| **Urine Color (1-8)** | Control | 0.386 | 0.004 | 4.30 (1.42) | 4.63 (1.28) | 0.536 |
|  | Hydration | 0.060 | 0.001 | 3.50 (2.17) | 3.37 (1.50) | 0.808 |
|  | Dehydration | 0.701 | 0.007 | 6.00 (0.82) | 5.56 (1.37) | 0.404 |
| **Urine Osmolality (mOsm/Kg)** | Control | 0.083 | 0.001 | 752.10 (303.84) | 726.37 (238.03) | 0.773 |
|  | Hydration | 0.003 | <0.001 | 460.30 (324.31) | 465.22 (295.37) | 0.956 |
|  | Dehydration | 1.258 | 0.012 | 923.20 (169.68) | 823.20 (169.68) | 0.265 |
| **Urine Specific Gravity** | Control | 0.293 | 0.003 | 1.02 (0.01) | 1.02 (0.01) | 0.589 |
|  | Hydration | 0.076 | 0.001 | 1.01 (0.01) | 1.01 (0.01) | 0.783 |
|  | Dehydration | 0.314 | 0.007 | 1.02 (0.00) | 1.02 (0.00) | 0.576 |
| **Hematocrit (%)** | Control | 25.991 | 0.198 | 50.65 (3.98) | 44.48 (3.45) | <0.001***** |
|  | Hydration | 26.596 | 0.202 | 50.68 (4.02)) | 44.44 (2.83) | <0.001***** |
|  | Dehydration | 29.696 | 0.220 | 50.83 (2.89) | 44.23 (3.05) | <0.001***** |
| **Hemoglobin (g/dl)** | Control | 31.365 | 0.230 | 16.57 (1.14) | 14.32 (1.19) | <0.001***** |
|  | Hydration | 32.689 | 0.237 | 16.52 (0.94) | 14.22 (1.06) | <0.001***** |
|  | Dehydration | 41.922 | 0.285 | 16.84 (0.66) | 14.24 (1.14) | <0.001***** |
| **Plasma Volume (mL)** | Control | 17.809 | 0.145 | 49.05 (4.20) | 55.37 (3.83) | <0.001^ |
|  | Hydration | 21.247 | 0.168 | 49.23 (4.33) | 56.14 (4.10) | <0.001^ |
|  | Dehydration | 27.936 | 0.210 | 48.95 (2.68) | 56.87 (4.43) | <0.001^ |
| **Plasma Volume Status** | Control | 20.825 | 0.166 | -23.08 (6.46) | -14.00 (5.29) | <0.001^ |
|  | Hydration | 21.126 | 0.167 | -23.08 (5.57) | -13.94 (5.15) | <0.001^ |
|  | Dehydration | 23.541 | 0.183 | -23.11 (4.43) | -13.80 (5.25) | <0.001^ |
| **Plasma Osmolality (mOsm/Kg)** | Control | 18.416 | 0.149 | 288.00 (3.65) | 282.78 (2.41) | <0.001***** |
|  | Hydration | 5.169 | 0.047 | 283.10 (5.70) | 280.33 (2.60) | 0.025***** |
|  | Dehydration | 9.300 | 0.081 | 288.60 (2.59) | 284.89 (3.59) | 0.003***** |
| **Serum Volume (mL)** | Control | 2.270 | 0.21 | 1.01 (0.25) | 1.10 (0.15) | 0.135 |
|  | Hydration | 4.610 | 0.042 | 0.94 (0.13) | 1.06 (0.13) | 0.034^ |
|  | Dehydration | 3.541 | 0.033 | 0.97 (0.19) | 1.08 (0.13) | 0.063 |
| **Serum Volume (g)** | Control | 8.136 | 0.072 | 1.31 (0.16) | 1.44 (0.12) | 0.005^ |
|  | Hydration | 13.412 | 0.113 | 1.24 (0.10) | 1.42 (0.15) | <0.001^ |
|  | Dehydration | 11.936 | 0.098 | 1.28 (0.11) | 1.44 (0.12) | 0.001^ |
| **Serum Osmolality (mOsmoles/Kg)** | Control | 5.081 | 0.098 | 284.00 (4.71) | 281.00 (3.22) | 0.026***** |
|  | Hydration | 3.773 | 0.035 | 280.40 (3.66) | 277.81 (3.59) | 0.055 |
|  | Dehydration | 2.778 | 0.026 | 285.00 (3.20) | 282.78 (3.61) | 0.098 |

**Supplementary Data Table 2:** Descriptive statistics for percent body water, serum osmolality, body mass, age, urine color, urine osmolality, urine specific gravity, hematocrit, hemoglobin, plasma volume, plasma volume status, plasma osmolality, serum volume in milliliters, and serum volume in grams between males and females among the control, hydration, and dehydration protocols. Data presented as mean (SD) for parametric data and median (Q1, Q3) for nonparametric data. ^#^ For the variable of age, a test statistic instead of F statistic was used as a Mann-Whitney U test was performed to compare the age of females to the age of males. * = significantly greater in males than females. ^ = significantly greater in females than males.
