# Supplementary material for: Plasma and serum volume remain unchanged following a 12-h fast from food and drink despite changes in blood and urinary hydration markers
Source: Eur J Clin Nutr. 2024 Oct 17;79(2):161–7. doi: 10.1038/s41430-024-01526-5 (PMC11810769; doi:10.1038/s41430-024-01526-5)
Supplement: Supplementary file 2 — Supplementary Table 3 [file 41430_2024_1526_MOESM2_ESM.pdf]

	A	B	C	D	E	F
1	Control	Body Weight (lbs)	Body Weight (kg)	%Body Water	Urine Color	Urine SG
2	02MC	173.8	79	57.2	4	1.017
3	05FC	107.6	48.90909091	56	4	1.021
4	06FC	167.8	76.27272727	43.7	4	1.014
5	07MC	157.8	71.72727273	57.4	5	1.025
6	08MC	171.2	77.81818182	61.4	2	1.004
7	09FC	138.2	62.81818182	51	6	1.022
8	10FC	131.8	59.90909091	56.1	3	1.004
9	11FC	136	61.81818182	52.6	7	1.026
10	12FC	139.6	63.45454545	52.2	4	1.019
11	13FC	138.2	62.81818182	52.2	6	1.026
12	14FC	130.4	59.27272727	55.1	5	1.022
13	15FC	139.8	63.54545455	55.7	4	1.019
14	16FC	133.4	60.63636364	55.5	4	1.024
15	17FC	127.6	58	53.5	4	1.026
16	18FC	125.2	56.90909091	56	4	1.024
17	19FC	134.4	61.09090909	55.8	4	1.022
18	20FC	140.6	63.90909091	54.7	4	1.023
19	21FC	119.8	54.45454545	55.2	6	1.03
20	22FC	115.3	52.40909091	57.1	4	1.026
21	23FC	125.2	56.90909091	unknown	5	1.024
22	25MC	177.8	80.81818182	61.2	5	1.027
23	26FC	133.6	60.72727273	51.9	7	1.025
24	27MC	142.2	64.63636364	58.6	5	1.023
25	30MC	204	92.72727273	53.7	7	1.027
26	31FC	174.2	79.18181818	50.8	6	1.017
27	32FC	166.6	75.72727273	52.9	4	1.011
28	34MC	146.4	66.54545455	64.2	3	1.013
29	35FC	132.6	60.27272727	50	4	1.011
30	36FC	125.6	57.09090909	54.7	3	1.013
31	37FC	115.6	52.54545455	53.7	5	1.023
32	39FC	144.2	65.54545455	53.4	2	1.006
33	40FC	144.6	65.72727273	53.3	7	1.023
34	41MC	171	77.72727273	62.7	5	1.023
35	43MC	189	85.90909091	63.4	4	1.016
36	44FC	175.6	79.81818182	44.8	4	1.013
37	45MC	185.8	84.45454545	60.6	3	1.009
38	46FC	131.8	59.90909091	54.4	5	1.02

	G	H	I	J	K
1	Urine Osmolality (mosm/kg)	Hct (%)	Hb (g/dl)	Plasma Vol. (mL)	Plasma Volume Status
2	614	46.5	15.2	53.5	-17.18873742
3	917	45.25	15	54.75	-10.2573211
4	541	43.25	13.7	56.75	-15.97059967
5	1023	54.25	17.8	45.75	-26.88117871
6	188	50.5	16.7	49.5	-23.00691948
7	821	43	15.1	57	-12.14307887
8	141	42	13.7	58	-9.63331563
9	841	41	13.2	59	-8.732205882
10	640	45.5	14	54.5	-16.18438754
11	948	48.25	14.9	51.75	-20.23516371
12	949	45	13.5	55	-14.09455521
13	717	42.25	13.2	57.75	-11.21433208
14	950	42.75	13.3	60.3	-11.04942185
15	936	44	14.5	56	-12.08482759
16	924	41	13	60.4	-6.953889776
17	815	39.75	13.4	60.25	-6.547946429
18	956	41	13.2	59	-9.406674964
19	1067	44.25	14.6	55.75	-11.12551857
20	861	46.75	15.6	53.25	-14.28655085
21	807	52.75	16.5	45.2	-25.48425919
22	1083	49	16.3	51	-21.62819071
23	699	50.5	16.1	49.5	-23.11716317
24	847	52.75	17.1	47.25	-21.64873418
25	1052	50.25	16.6	49.75	-26.65064103
26	602	40	12.8	60	-14.74278962
27	420	42.5	12.8	54	-11.7826062
28	538	52	16.7	45	-21.2408575
29	403	42.25	13.4	57.75	-10.1484474
30	484	47.25	15.6	52.75	-16.87423169
31	785	45.5	15.7	54.5	-12.33278979
32	251	52.75	16.8	47.25	-27.84725121
33	930	44.5	15.3	54.9	-15.29974585
34	992	42.25	14.3	57.75	-10.14068826
35	761	53.25	16.8	46.75	-29.50396825
36	497	44.25	14.3	53.5	-18.15258685
37	423	55.75	18.2	44.25	-32.92578869
38	710	43.75	13.4	56.25	-12.35989662

	L	M	N
1	Plasma Osmolaltiy (mosm/kg)	Serum volume (g)	Serum volume (mL)
2	291	1.477	1.6
3	285	1.342	1.2
4	279	1.45	1.2
5	288	1.138	1
6	290	1.328	1.1
7	282	1.474	1
8	283	1.5	1.2
9	288	1.528	1.2
10	282	1.421	1
11	287	1.341	1
12	284	1.337	1
13	285	1.563	1.2
14	283	1.534	1.2
15	285	1.478	1
16	279	1.606	1.2
17	281	1.572	1.2
18	281	1.533	1.2
19	282	1.504	1.2
20	288	1.324	1
21	282	1.214	0.8
22	289	1.317	1
23	280	1.316	1
24	287	1.203	0.8
25	287	1.277	0.8
26	283	1.641	1.4
27	281	1.566	1.2
28	286	1.167	0.8
29	280	1.421	1.2
30	281	1.382	1
31	283	1.331	1
32	282	1.259	0.8
33	282	1.467	1.2
34	294	1.643	1.2
35	288	1.394	1
36	283	1.268	0.8
37	280	1.138	0.8
38	284	1.594	1.2

	O	P	Q	R
1	Serum osmolality (mosm/kg)		Hydration	Body Weight (lbs.)
2	288		02MH	172.2
3	283		05FH	107.6
4	277		06FH	170.8
5	278		07MH	158
6	287		08MH	169
7	281		09FH	138.4
8	279		10FH	132.8
9	283		11FH	136
10	279		12FH	140.6
11	286		13FH	137.8
12	274		14FH	131
13	285		15FH	141.8
14	288		16FH	135
15	283		17FH	127.6
16	278		18FH	124.6
17	280		19FH	134.4
18	280		20FH	140.6
19	278		21FH	119.2
20	284		22FH	114.8
21	281		23FH	126.2
22	286		25MH	175.2
23	280		26FH	134.4
24	282		27MH	142
25	288		30MH	207.4
26	283		31FH	176.4
27	278		32FH	166.6
28	283		34MH	143.6
29	278		35FH	131.8
30	280		36FH	126.2
31	287		37FH	116.4
32	280		39FH	144
33	279		40FH	142.8
34	291		41MH	169.4
35	277		43MH	189
36	281		44FH	176.8
37	280		45MH	187
38	282		46FH	131.8

	S	T	U	V	W
1	Body Weight (kg)	%Body Water	Urine Color	Urine SG	Urine Osmolality (mosm/kg)
2	78.27272727	56.3	3	1.016	628
3	48.90909091	59.9	1	1.001	98
4	77.63636364	43.4	4	1.02	667
5	71.81818182	56.3	6	1.023	816
6	76.81818182	60.5	1	1.002	108
7	62.90909091	54.2	3	1.008	325
8	60.36363636	56	3	1.005	207
9	61.81818182	52.8	4	1.014	492
10	63.90909091	52.5	3	1.004	177
11	62.63636364	51.9	4	1.021	845
12	59.54545455	54.6	4	1.018	658
13	64.45454545	56.1	1	1.001	71
14	61.36363636	54.9	2	1.002	98
15	58	53.3	4	1.025	898
16	56.63636364	56.9	6	1.025	882
17	61.09090909	56.3	4	1.019	584
18	63.90909091	55.4	4	1.026	935
19	54.18181818	54.8	2	1.004	152
20	52.18181818	58.1	7	1.021	643
21	57.36363636	53.2	3	1.01	366
22	79.63636364	61.6	2	1.002	115
23	61.09090909	52.5	3	1.016	726
24	64.54545455	60.9	4	1.021	810
25	94.27272727	53.8	6	1.019	708
26	80.18181818	49.8	3	1.007	262
27	75.72727273	52.8	2	1.002	97
28	65.27272727	63.4	2	1.003	144
29	59.90909091	50.3	3	1.009	319
30	57.36363636	54.6	5	1.02	662
31	52.90909091	53	4	1.017	615
32	65.45454545	54.4	6	1.023	808
33	64.90909091	52.6	1	1.003	132
34	77	63	7	1.023	805
35	85.90909091	52.1	3	1.01	383
36	80.36363636	45.1	2	1.002	142
37	85	61.3	1	1.001	86
38	59.90909091	53.9	3	1.02	700

	X	Y	Z	AA	AB
1	Hct (%)	Hb (g/dl)	Plasma Vol. (mL)	Plasma Volume Status	Plasma Osmolaltiy (mosm/kg)
2	45	15.2	55	-14.61315108	287
3	39.75	13.8	65.48913043	-1.242074814	277
4	47.5	15.3	47.00980392	-22.52469262	279
5	52	17.2	49.6744186	-23.31840312	288
6	49.25	16.5	51.4	-20.72959338	288
7	44.5	14.3	58.6048951	-14.48268064	278
8	44.5	13.5	56.32222222	-13.67911145	280
9	44.75	14.1	51.72340426	-14.533125	287
10	43.5	13.7	57.73722628	-13.24537518	281
11	47.25	14.1	55.7429078	-18.64116382	282
12	47	13.3	53.79699248	-17.30685115	279
13	44.75	13.5	54.02222222	-15.32275123	281
14	43.5	14	56.5	-12.45325	279
15	44.5	14.4	55.88541667	-12.86978448	283
16	40	13.3	60	-5.26717496	278
17	39	13.6	60.10294118	-5.384642857	279
18	40.75	13.7	57.08759124	-9.022804943	282
19	42	13.4	63.19402985	-7.422852349	276
20	44.75	14.6	59.03424658	-10.96809016	285
21	48.75	15.8	51.25	-19.33018522	279
22	50.5	16.7	48.31437126	-23.57665964	280
23	45.75	15.1	57.84271523	-15.854375	280
24	57	17.6	41.77840909	-28.65944384	287
25	51	17.1	47.56725146	-28.09625893	285
26	42	12.3	60.35772358	-14.9205102	280
27	41.5	12.8	58.5	-13.26005552	279
28	55.5	17.1	43.5	-26.47218056	282
29	43.5	14	54.1	-11.97038505	277
30	47	15.5	53	-16.57560618	283
31	47.75	16.08	52.25	-16.09969716	284
32	50.5	17.1	48.6	-24.38875	282
33	45	15	56	-15.83497899	282
34	44	15	53.4	-12.5967366	287
35	50.5	15.5	53.7	-25.35714286	273
36	45	13.9	56.6	-19.35469457	278
37	52	17.3	48	-27.38461538	274
38	45	13.8	55	-14.30745448	279

	AC	AD	AE	AF
1	Serum volume (g)	Serum volume (mL)	Serum osmolality (mosm/kg)	
2	1.3	1.2	282	
3	1.616	1.2	277	
4	1.344	1.1	274	
5	1.256	1	279	
6	1.205	1	284	
7	1.403	1	273	
8	1.509	1.2	279	
9	1.518	1.2	285	
10	1.497	1.2	283	
11	1.316	1	278	
12	1.339	1	276	
13	1.501	1.2	283	
14	1.464	1	277	
15	1.574	1.2	278	
16	1.513	1.2	275	
17	1.61	1	276	
18	1.577	1.2	280	
19	1.539	1.2	271	
20	1.284	1	285	
21	1.232	0.8	279	
22	1.225	0.8	277	
23	1.301	1	277	
24	1.15	0.8	279	
25	1.231	0.8	285	
26	1.588	1.2	278	
27	1.227	0.8	278	
28	1.148	0.8	280	
29	1.503	1	276	
30	1.236	1	279	
31	1.288	1	283	
32	1.26	1	277	
33	1.093	0.8	277	
34	1.505	1	284	
35	1.2435	1	273	
36	1.3537	1	274	
37	1.1758	1	281	
38	1.58	1.2	273	

	AG	AH	AI	AJ	AK	AL
1	Dehydration	Body Weight (lbs)	Body Weight (kg)	%Body Water	Urine Color	Urine SG
2	02MD	171.4	77.90909091	56.5	7	1.022
3	05FD	106.8	48.54545455	57.6	5	1.021
4	06FD	168.6	76.63636364	44	4	1.014
5	07MD	157	71.36363636	56.29	7	1.029
6	08MD	169.6	77.09090909	61.1	6	1.019
7	09FD	137.8	62.63636364	52.6	6	1.02
8	10FD	130.2	59.18181818	56.6	7	1.021
9	11FD	135	61.36363636	52.3	6	1.024
10	12FD	139.6	63.45454545	52.4	5	1.017
11	13FD	140.2	63.72727273	52.7	4	1.02
12	14FD	128.8	58.54545455	55.5	7	1.029
13	15FD	139.2	63.27272727	55.5	3	1.015
14	16FD	135.4	61.54545455	57.8	5	1.027
15	17FD	125	56.81818182	53.9	7	1.027
16	18FD	124.6	56.63636364	54.5	7	1.027
17	19FD	133	60.45454545	56.5	4	1.021
18	20FD	139.8	63.54545455	54.5	4	1.022
19	21FD	116.8	53.09090909	54.9	4	1.02
20	22FD	116	52.72727273	57.1	5	1.018
21	23FD	126.4	57.45454545	53.4	7	1.026
22	25MD	176.8	80.36363636	62.1	5	1.028
23	26FD	132.2	60.09090909	52.2	7	1.019
24	27MD	140.2	63.72727273	57	5	1.021
25	30MD	205	93.18181818	54.1	7	1.025
26	31FD	174.8	79.45454545	50.5	6	1.023
27	32FD	166.2	75.54545455	52	5	1.023
28	34MD	143	65	63.3	6	1.025
29	35FD	130	59.09090909	49.8	7	1.022
30	36FD	125.2	56.90909091	55.3	7	1.023
31	37FD	116.4	52.90909091	53.5	3	1.019
32	39FD	141.6	64.36363636	53.6	7	1.026
33	40FD	142.4	64.72727273	53.5	7	1.026
34	41MD	167.4	76.09090909	62.1	6	1.022
35	43MD	186.4	84.72727273	53.4	5	1.026
36	44FD	174	79.09090909	45.5	5	1.02
37	45MD	185.6	84.36363636	60.7	6	1.018
38	46FD	130.4	59.27272727	53.9	6	1.026

	AM	AN	AO	AP	AQ
1	Urine Osmolality (mosm/kg)	Hct (%)	Hb (g/dl)	Plasma Vol. (mL)	Plasma Volume Status
2	853	46.5	16.2	50.19753086	-16.816728
3	884	44.25	14.8	56.50337838	-8.433757022
4	599	45	13.5	55.81481481	-18.63572064
5	1067	50.25	16.9	52.39940828	-20.34962437
6	727	50.5	17.1	48.3	-22.77149855
7	882	43.75	14.6	58.17636986	-13.24294721
8	718	42.75	14.3	54.8479021	-10.54819412
9	860	41.75	13.3	57.81203008	-9.741625
10	681	41.5	13.8	59.34782609	-10.03278295
11	875	43	14	60.66428571	-12.42267118
12	1003	44.25	12.8	58.79882813	-12.67074146
13	618	46.25	14	50.67857143	-17.28523707
14	881	40.5	12.3	67.7	-7.866622969
15	859	46	14.8	52.90540541	-14.80636
16	958	41.5	13.7	56.7	-7.635495586
17	658	39.5	13.4	60.5	-5.935009398
18	804	38.75	13.4	60.3358209	-5.833382511
19	725	42.5	14.3	58.70629371	-7.749914384
20	763	43	14.1	63.06382979	-8.392155172
21	910	46.5	15.1	60	-15.82045886
22	1213	50	16.4	49.69512195	-23.02761341
23	782	51.25	16.5	47.56818182	-24.09842568
24	848	53.75	17.4	45.45258621	-22.90651633
25	903	51	16.9	48.13017751	-27.85753596
26	888	44	12.6	56.88888889	-17.71620137
27	879	46	13.6	54	-19.89528881
28	1044	55	17.6	45	-25.53254438
29	728	46	14.2	60	-15.59592308
30	748	45.25	15.1	56.2	-13.65636382
31	868	45	16.1	53.6	-11.68389175
32	899	52.5	17	46.9	-27.17807203
33	985	44	15.6	56	-14.25235955
34	860	47	15.5	53	-16.95643783
35	1056	49.75	16.8	50.25	-23.90611588
36	837	46.5	14.4	53.5	-21.32271552
37	661	54.5	17.6	47.1	-31.00826149
38	935	43	13.1	58.3	-10.97072086

	AR	AS	AT
1	Plasma Osmolaltiy (mosm/kg)	Serum volume (g)	Serum volume (mL)
2	291	1.418	1.4
3	284	1.397	1
4	281	1.392	1.1
5	287	1.221	1
6	290	1.342	1
7	283	1.436	1
8	282	1.462	1
9	289	1.474	1.2
10	285	1.49	1.2
11	283	1.435	1
12	286	1.368	1
13	291	1.457	1
14	287	1.653	1.4
15	286	1.481	1
16	281	1.567	1.2
17	279	1.645	1.2
18	280	1.587	1.2
19	285	1.521	1.2
20	288	1.585	1.2
21	283	1.392	1
22	285	1.278	0.8
23	279	1.23	1
24	285	1.139	0.8
25	291	1.328	1
26	288	1.405	1
27	284	1.272	1
28	291	1.135	0.8
29	282	1.332	1
30	285	1.394	1
31	293	1.474	1
32	289	1.23	0.8
33	286	1.283	1
34	289	1.478	1.1
35	291	1.259	0.8
36	289	1.429	1.2
37	286	1.244	1
38	284	1.596	1.2

	AU	AV
1	Serum osmolality (mosm/kg)	Age (yrs)
2	283	20
3	281	21
4	279	21
5	281	20
6	289	21
7	284	20
8	277	20
9	287	20
10	279	21
11	282	20
12	283	20
13	293	19
14	286	18
15	285	20
16	280	19
17	283	18
18	278	20
19	278	19
20	285	20
21	285	20
22	280	18
23	280	21
24	285	18
25	289	21
26	285	20
27	285	20
28	286	21
29	279	21
30	283	21
31	288	21
32	284	21
33	280	20
34	286	20
35	288	22
36	284	20
37	283	20
38	282	20

Supplementary Table 3: Raw data used for statistical analyses. The “unknown” in %Body Water was the subject who forgot to record their %Body Water and the multiple imputation approach was used to account for the missing data. SG=specific gravity; Hct = hematocrit; Hb = hemoglobin; Vol. = volume
